# Influence of remaining coronal thickness and height on biomechanical behavior of endodontically treated teeth: survival rates, load to fracture and finite element analysis

**DOI:** 10.1590/1678-7757-2017-0313

**Published:** 2018-04-18

**Authors:** Gislene Corrêa, Lucas P Brondani, Vinícius F. Wandscher, Gabriel K. R. Pereira, Luiz F. Valandro, César D. Bergoli

**Affiliations:** 1 Universidade Federal de Pelotas, Programa de Pós-Graduação em Odontologia, Pelotas, Rio Grande do Sul, Brasil.; 2 Universidade Federal de Santa Maria, Programa de Pós-Graduação em Odontologia, Santa Maria, Rio Grande do Sul, Brasil.; 3 Faculdade Meridional - IMED, Programa de Pós-Graduação em Odontologia, Passo Fundo, Rio Grande do Sul, Brasil.; 4 Universidade Federal de Santa Maria, Faculdade de Odontologia, Santa Maria, Rio Grande do Sul, Brasil.

**Keywords:** Crown, Finite element analysis, Nonvital tooth

## Abstract

**Objective:**

To evaluate the effect of restorative strategy (fiber post vs cast post and core), coronal height (0 mm vs 2 mm) and thickness (higher than 1 mm vs lower than 1 mm) on survival rate, fracture resistance and stress distribution.

**Material and Methods:**

Seventy-two bovine teeth were cleaned and allocated in six groups (n = 12). Twenty-four teeth were sectioned at 13 mm length (no remaining coronal structure) and forty-eight were sectioned at 15 mm (2 mm remaining coronal structure). Half of the forty-eight had remaining coronal thickness lower than 1 mm and the other half had thickness higher than 1 mm. All root canals were prepared at 10 mm (luting length), fiber posts were cemented in thirty-six specimens and cast post and core in other thirty-six. All teeth were restored with metallic crowns. Specimens were submitted to 1.5 million cycles (100 N, 45°, 10 Hz at 2 mm below incisal edge) and evaluated at each 500,000 cycles to detect failures. Specimens that survived were submitted to load to fracture test. Bidimensional (Rhinoceros^®^ 4.0) models were obteined survival data submitted to Kaplan-Meier (α=0.05) analysis and load to fracture values submitted to ANOVA and Tukey tests (α=0.05).

**Results:**

Groups without remaining coronal structure showed survival rates lower than other groups (p=0.001). ANOVA showed higher values of load to fracture for groups with coronal thickness higher than 1 mm (p=0.0043). Finite element analysis showed better stress distribution in groups with remaining coronal structure and restored with fiber post.

**Conclusion:**

Specimens without remaining coronal structure have lower survival rates. Specimens with remaining structure lower than 1 mm and without coronal structure support the same load to fracture value independently of the restorative strategy.

## Introduction

The reconstruction of endodontically treated teeth with reduced remaining coronal structure is usually associated with intracanal posts to provide retention and mechanical strength to coronal restoration^5^ ^,^ ^22^. This can be related to the fact that teeth with no remaining coronal structure may have lower fracture resistance and increased risk of catastrophic fracture^10^. In contrast, the presence of coronal structure could improve the biomechanical behavior of the system, reducing the stress transmission to the root and increasing the fracture strength of these teeth^14^ ^,^ ^25^. Besides, some authors have reported that with remaining coronal structure the tensile stresses are redistributed at the outer surface of the cervical third of the root, decreasing the possibility of a catastrophic failure. On the other hand, when there is no coronal structure, the stress distribution is different, increasing the possibility of irreversible fracture^12^ ^,^ ^17^ ^,^ ^20^.

Many post systems are available for restoration of endodontically treated teeth. Among them, the cast post and core have been used for many years and can be considered as gold standard strategy. However, it has some limitations, such as the need of at least two clinical sessions to be obtained, the need for greater dentin removal and a high elastic modulus (200 GPa) that increases the chance of catastrophic fractures^18^ ^,^ ^19^. Currently, glass fiber posts have been used to restore endodontically treated teeth, mainly because they have an elastic modulus (30-50 GPa) very similar to dentin^19^, are more aesthetic and demand fewer operative steps for their manufacture. These positive characteristics are reflected on the results of clinical studies, which have shown high survival rates of teeth restored with glass fiber posts^8^ ^,^ ^11^ ^,^ ^18^.

Clinical studies provide high level of scientific evidence about the quality and behavior of a material or restorative technique. However, these studies are time consuming, difficult to implement and expensive^1^. Thus, laboratory tests are important assessment tools because they allow a comparison of different materials under controlled conditions^1^. Aging protocols simulating clinical parameters as load, temperature and humidity, are important to better predict the behavior of a material or restorative strategy. At this scenario, the mechanical cycling almost reproduces those parameters and may induce the propagation of pre-existing cracks and fractures through low intensity forces^17^ ^,^ ^26^.

It is very important to comprehend the biomechanical behavior of endodontically treated teeth to clarify the reasons/factors that lead to its failure. Thus, the use of non-destructive tests, such as finite element analysis, associated with laboratory testing, allows a better understanding of the phenomena that occur, in addition to the behavior of the tested system^15^ ^,^ ^21^.

As previously mentioned, several factors must be considered to choose the best strategy to restore endodontically treated tooth. However, there is no scientific evidence regarding the thickness of the remaining coronal structure and how it can influence on the survival of endodontically treated teeth.

Thus, this study aimed to evaluate the influence of different retainers, remaining coronal thickness and remaining coronal height on: (1) survival rates of endodontically treated teeth subjected to mechanical cycling; (2) load to fracture of endodontically treated teeth and (3) stress distribution through finite element analysis. Considering the scientific assumptions related to this issue, our hypotheses were that different restorative strategies, height and thickness of the remaining coronal structure showed different values of survival rate, load to fracture and stress distribution in endodontically treated teeth.

## Material and methods

### Selection, preparation and randomization of specimens

Initially, the sample size was calculated using a standard deviation of 150 N, with statistically significant difference of 200 N between groups^23^, conferring a significance level of 5%, the power sample was 95% and the hypothesis test was considered one-tailed. Based on these criteria, the sample size estimated was 12 specimens *per* group.

Seventy-two bovine incisors were selected for the study. Initially they were cleaned with scalpel blade no. 11, and analyzed by a magnifying glass (4x) (EyeMag^®^ Pro, Carl Zeiss of Brazil Ltda, São Paulo, SP, Brazil), to detect any fracture/fissure or defect that could interfere on the results. The teeth were stored for 2 hours in 1.23% chlorhexidine for disinfection and stored in a humid environment under refrigeration (distilled water at 4°C) until used in the study. After selection, the coronal portion of the teeth was sectioned with a diamond no. 3216 (KG Sorensen, Indústria e Comércio Ltda, Cotia, SP, Brazil) using a high-speed handpiece with water cooling. Forty-eight teeth were sectioned in 15 mm (2 mm of remaining coronal dentin) and twenty-four teeth in 13 mm (with no remaining coronal dentin). To standardize the sample, the diameter of the canal was used as inclusion factor. For this, the mesiodistal and buccolingual diameter of root canal were measured with a digital caliper (Black Jack India Ltda., Gurgaon, Haryana, India) and if one of the diameters exceed 2 mm (corresponding to the diameter of the glass fiber pin White Post DC # 3, FGM, Joinville, SC, Brazil) the specimen was replaced.

After sample selection, root canals were prepared with NiTi instruments (Maillefer, Dentsply Ind. e Com. Ltda., Petrópolis, RJ, Brazil), associated with 2.5% sodium hypochlorite irrigation, and obturated using gutta-percha cones (Maillefer, Dentsply Indústria e Comércio Ltda., Petrópolis, RJ, Brazil) and calcium hydroxide base cement (Sealer 26, Dentsply Indústria e Comércio Ltda., Petrópolis, RJ, Brazil) by lateral condensation technique. Subsequently, periodontal ligament (PDL) and embedding procedures were performed according with methodology described by Wandscher, et al.^23^ (2014).

Specimens were prepared to receive a metallic crown (chamfer with 1.2 mm thickness), using the diamond bur 4138 (KG Sorensen, Medical Burs Ind. e Com. Tips and Drills Surgical Ltda, Cotia, SP, Brazil) in high speed with air-water irrigation. The drills were changed every 5 preparations. After coronal preparation of specimens, the remaining coronal tissue thickness was measured with a digital caliper and the specimens were allocated in different groups (remaining coronal dentin higher than 1 mm thickness; remaining coronal dentin lower than 1 mm thickness).

**Figure 1:**
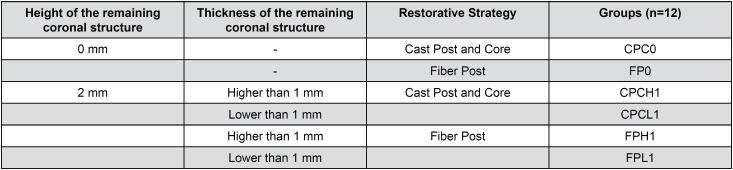
Study design

Finally, specimens were divided into 6 experimental groups, according to the remaining coronal height, thickness and restorative strategy (Figure 1). Specimens with coronal structure greater than 1 mm, less than 1 mm and samples without coronal structure were numbered from 1 to 24 and three random number sequences of 24 numbers were generated by the computer program Random Allocator.

### Restorative strategies

#### Cast post and core

All canals were prepared with the custom bur of the fiber post system (White Post DC #3 FGM, Joinville, SC, Brazil). In the groups without remaining coronal structure this preparation had 10 mm length and in the other groups the preparation had 12 mm length, always keeping a length of 3 mm of filling material. The canals were modeled using a prefabricated plastic post (Pinjet, Ângelus, Londrina, PR, Brazil) and acrylic resin (Duralay; Reliance Dental Manufacturing Company, Chicago, IL, USA) followed by casting, as recommended by the manufacturer (NiCr alloy, Wironia Light, Bego, Bremen, Germany). The core was standardized using plastic matrices.

The Cast Post and Core groups (CPC) were evaluated for adaptation with liquid carbon and cemented as follows: the post was cleaned with isopropyl alcohol and air-abraded with aluminum oxide particles (50 μm pressure: 2.8 bar, 10 mm distance for 15 seconds), root dentin and/or coronal dentin was conditioned with 37% phosphoric acid (Condac 37, FGM, Joinville, SC, Brazil) for 15 seconds, washed with water for 15 seconds, dried with paper points (Dentsply Indústria e Comércio Ltda, Petrópolis, RJ, Brazil), a photo-cured adhesive agent (Ambar, FGM, Joinville, SC, Brazil). was applied with a microbrush, the excess was removed with paper cones and photo activated for 20 s with high power LED (KaVo Poly Wireless, KaVo, São Paulo, SP, Brazil), dual resin cement (Allcem, FGM, Joinville, SC, Brazil) was inserted into the canal with the aid of mixing tips/applicator system, posts were positioned, excess of cement was removed and photo activation was performed in the cervical portion of the root for 40 seconds.

### Prefabricated fiber post

Initially, fiber posts (White Post DC #3, FGM, Joinville, SC, Brazil) were positioned in the canal and sectioned at 15 mm length, corresponding to 5 mm height of coronal portion. Then, fiber posts were cleaned with 70% alcohol and received the application of a MPS-based silane coupling agent (Prosil, FGM, Joinville, SC, Brazil). Cementation was carried out identically to cast post and core groups.

After cementation, the core was performed with hybrid composite resin (Oppalis, FGM, Joinville, SC, Brazil) using the same plastic matrices in CPC groups. A layer of resin was inserted into the matrix, which was placed over the post and the tooth surface. The photo-activation was performed for 20 seconds on each face and the matrices were removed with a probe.

### Preparation and cementation of crowns

Seventy-two metallic crowns were made according to the anatomy of an upper central incisor with standard height dimensions of 10 mm and mesio-distal dimension of 8.5 mm^9^. For the confection of crowns, the impression of each teeth was performed (Express XT, 3M ESPE, Saint Paul, MN, USA) and a master die was obtained with type IV dental stone (Durone, Dentsply Indústria e Comércio Ltda, Petrópolis, RJ, Brazil). Each crown was waxed on a die (Newwax, Technew, Campo Grande, MS, Brazil) using standardized maxillary incisive plastic matrices. The waxed crowns were sent to a commercial laboratory and fused according to the manufacturer's recommendation, with the same alloy used to obtain the cast post and core.

The crowns were examined for adaptation and then abraded with 50 μm aluminum oxide particles (Microjato, Bio-Art Equipamentos Odontológicos Ltda, São Carlos, SP, Brazil). For luting, the coronal structure was conditioned with 37% phosphoric acid for 20 seconds, followed by rinsing and drying with absorbent paper. After that, adhesive system (Ambar, FGM, Joinville, SC, Brazil) was applied and photoactivated during 20 s with high-power LED (KaVo Poly Wireless, KaVo, São Paulo, SP, Brazil). Finally, the dual resin cement (Allcem, FGM, Joinville, SC, Brazil) was inserted into the crown and positioned; excesses were removed and the photo-activation of each face was made for 40 seconds.

### Mechanical cycling

For mechanical cycling in the fatigue machine (Instron Electropuls E3000, Instron, Norwood, MA, USA), the specimens were subjected to the following protocol: angle of 45°, water immersion at ±37°C, load pulses of 0 to 100 N, frequency of 10 Hz and 1,500,000 cycles at a point located 2 mm below the incisal edge on the lingual surface. For survival analysis, roots were evaluated at each 500,000 cycles by a trained and blinded operator. Once having one of the outcomes, the number of cycles was recorded and the cycling was interrupted. Failures were examined under magnification of 50x with a stereomicroscope (Discovery V20, Carl Zeiss Microscopy, Göttingen, NI, Germany).

### Load to fracture

Specimens that survived to mechanical cycling were subjected to load to fracture test in a universal testing machine (DL-1000, Emic, São José dos Pinhais, PR, Brazil) at a 45° inclination and cross head speed of 1 mm/min until failure.

### Failure analysis

Using a stereomicroscope under 7.5x to 50x magnifications (Discovery V20, Carl Zeiss Microscopy, Göttingen, NI, Germany) all fractured specimens were classified into different modes: favorable/restorable (root fracture above the simulated PDL, adhesive failure of the crown, dislodgment of crown/core, adhesive failure of the post); unfavorable (root fracture below the simulated PDL).

### Finite element analysis

Six two-dimensional models were obtained by design software (Rhinoceros 4.0). All parameters (root length, periodontal ligament, dimensions of the post, acrylic resin and metal crown) were simulated in an identical scenario to those used in the laboratory test. The dimensions of vestibular-lingual roots, the thickness of the remaining coronal structure (Ø=0.8 mm to groups Cast Post and Core lower than 1 mm - CPCL1 and Fiber Post lower than 1 mm - FPL1; Ø=1.2 mm to groups Cast Post and Core higher than 1 mm - CPCH1 and Fiber Post higher than 1 mm - FPH1) and the dimensions of the cores were obtained based on measurements with a digital caliper of specimens from each group. The thickness of resin cement between the post and the root dentin and between the crown and the core was determined as 0.1 mm.

**Figure 2:**
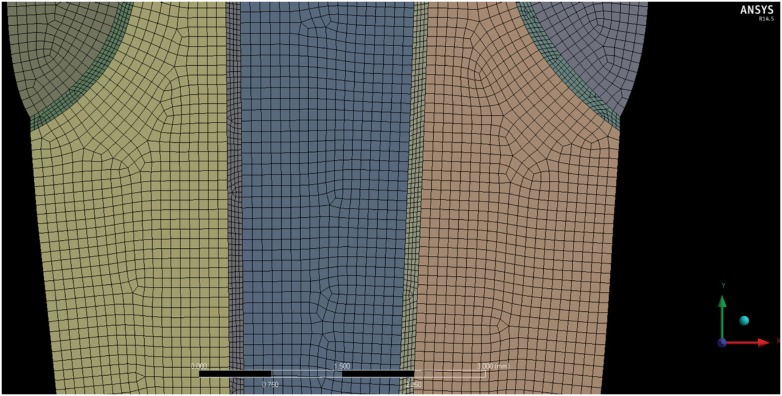
The image above shows the mesh created for the models, with a tetrahedral element format and approximately 37,000 elements and 188,000 nodes

**Figure 3:**
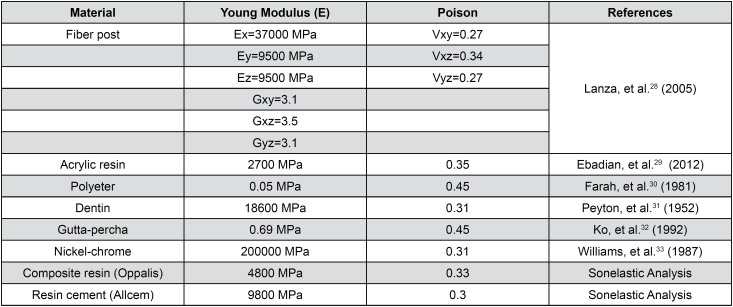
Materials, properties and references used for the finite element analysis

After modeling, geometries were imported to a post-processing software (ANSYS 13.0, Canonsburg, PA, USA), using the *stp* format, for meshing and apply boundary conditions. After convergence tests, the element size was determined in 0.1 mm, except for resin cement layers, which were refined until a size of 0.05 mm, with a tetrahedral format, resulting in a mesh with approximately 37,000 elements and 118,000 nodes (Figure 2). The interfaces were considered bonded and the edges of acrylic resin were considered fixed on the axes x, y and z. A 100 N force was applied at a point located 2 mm bellow the incisal edge on the palatal face, with a 45° angle. The fiber posts were considered orthotropic and other materials isotropic. The properties of the resin cement (Allcem, FGM, Joinville, SC, Brazil) and composite (Oppalis, FGM, Joinville, SC, Brazil) used in the studies were obtained by the natural wave propagation method in accordance with ASTM standard E-1876 (2007), through the apparatus Sonelastic^®^ (ATCP Physical engineering, São Carlos, SP, Brazil), while the properties of other materials were obtained in the literature (Figure 3). All materials were homogeneous and linear. Values of maximum principal stress (σ1) were analyzed at the root and at the resin core material.

### Statistical analysis

Survival rates were calculated using the KaplanMeier and Log-rank test (α=0.05). Load to fracture was subjected initially to normality and homoscedasticity (Anderson-Darling) tests and the values were compared using one-way ANOVA followed by Tukey test (α=0.05).

## Results

### Analysis of survival data (Kaplan-Meier method)

The Kaplan-Meier test showed significant differences among groups (p = 0.000). The group Cast Post and Core - CPC0 (survival rate of 0.75) and group Fiber Post - FP0 (survival rate of 0.83) had values lower than other groups after 1.5 milion cycles. There were only 5 failures during mechanical cycling, three specimens of group CPC0 (unfavorable fractures in 108,272, 498,000 and 532,177 cycles) and two specimens from group FP0 (an unfavorable fracture in 175,508 cycles and adhesive failure of the post in 1,025,067 cycles).

### Load to fracture

The one-way ANOVA showed a statistical difference between groups (p=0.0043). Table 3 shows the mean values and standard deviations of the studied groups after comparison by Tukey test.

### Failure analysis

**Figure 4:**
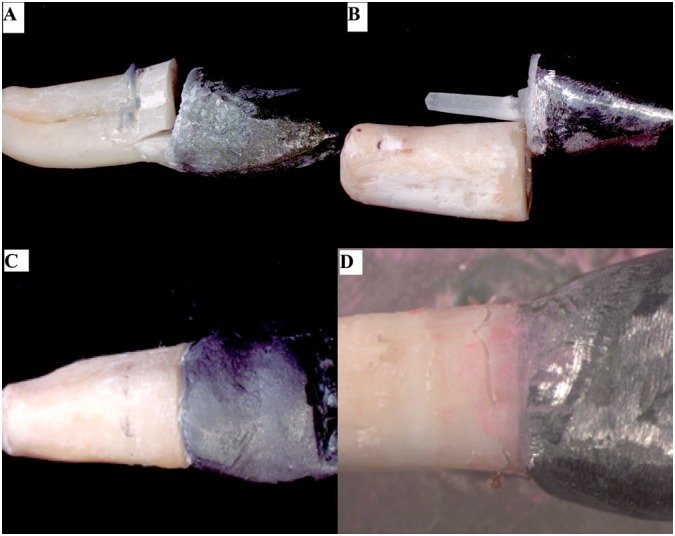
Representative images of failures during mechanical cycling. (A): Unfavorable fracture of specimen without remaining coronal structure. (B): Adhesive failure of the post. (C): Adhesive failure of the crown (displacement crown/core/post); (D) Fracture favorable of the remaining tooth structure

**Figure 5:**
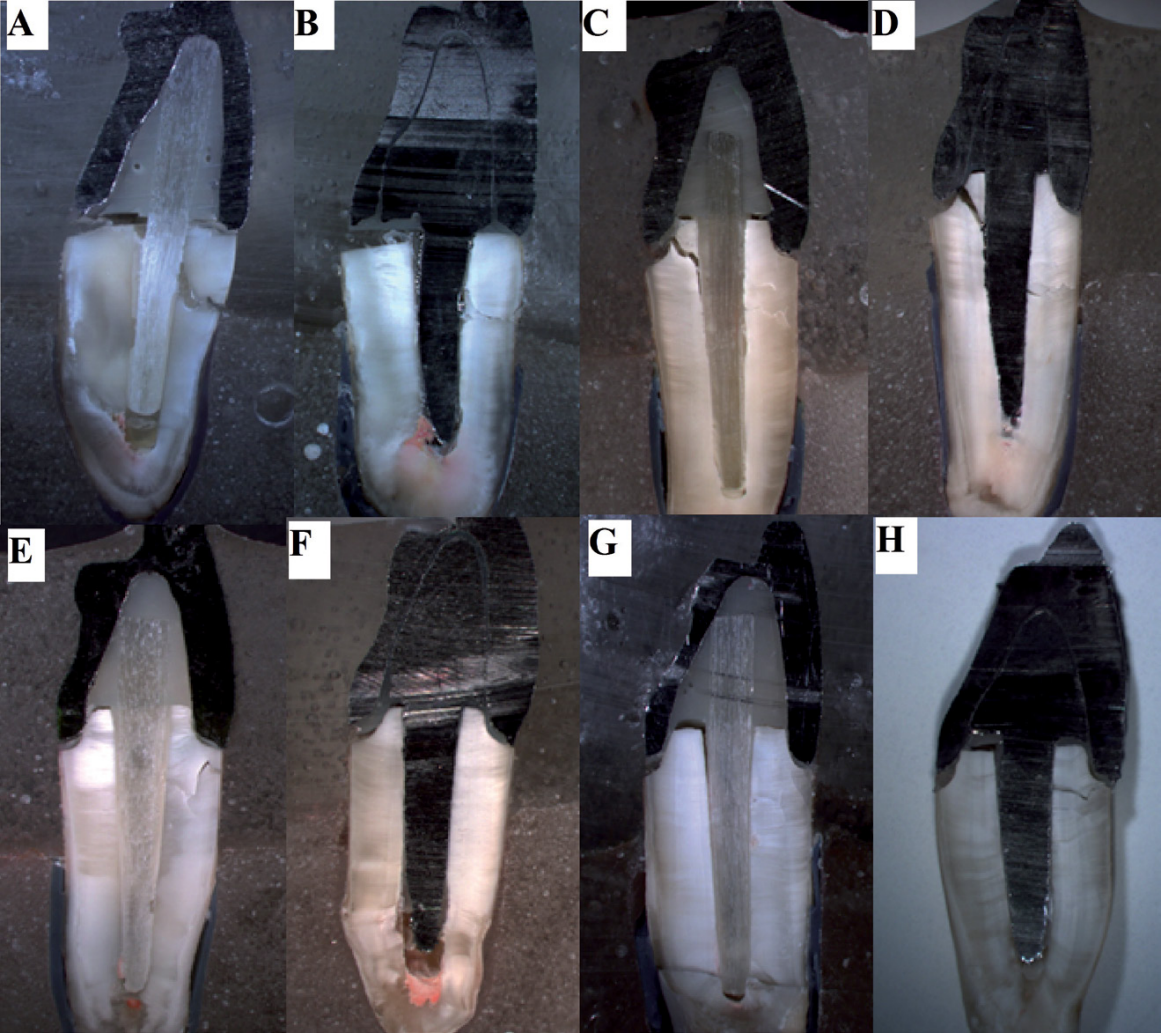
Representative fractures evaluated under stereomicroscope 7.5x. Roots A and B restored with fiber post and cast post and core, respectively without remaining coronal structure with displacement of the crown/core/post assembly and buccal fracture. Roots C and D restored with fiber post and cast post and core, respectively with remaining coronal structure lower than 1 mm with displacement of the crown/core/post assembly, cracks on the lingual surface of the coronal remaining and partial buccal fracture. Root E restored with fiber post with displacement of the crown/core/post assembly and total buccal fracture. Root F shows restoration with post and core with displacement of the crown/core/post assembly, both E and F roots do not have cracks on the lingual surface of the dental remaining. G and H roots restored with fiber post and cast post and core, respectively with remaining coronal structure higher than 1 mm with displacement of the crown/core/post assembly and partial (G) and total (H) buccal fracture

The most prevalent failures were unfavorable root fractures. Dislodgement of the crown/core and root fracture above periodontal ligament were the most predominant modes between favorable failures (Table 1, Figure 4). All fractures were observed in stereomicroscope (Discovery V20, Carl Zeiss) and coronal portions of representative specimens were embedded in acrylic resin and sectioned in the buccolingual sense to observe the internal cracks (Figure 5).

**Table 1:** Distribution of the failure modes occurred during mechanical cycling and after load to fracture test. Mean and standard deviation (Newton) of values for fracture

Groups	Failure mode				
		Favorable		Unfavorable	
	Root fracture (above ligament)	Adhesive failure of the post	Dislodgement of crown/core	Root fracture (below ligament)	Mean (SD)*
Group CPC0	6			6	388.17B (103.06)
Group FP0	2	1	3	6	410.68B (130.24)
Group PCH1	3			9	681.86A (160.44)
Group PCL1	3		4	5	514.44B (53.008)
Group FPH1	2		7	3	711.81A (195.92)
Group FPL1	2		4	6	433.79B (110.59)
Total	18 (25%)	1 (1%)	18 (25%)	35 (49%)	

* Different letters indicate significant differences between the groups

### Data analysis by finite element method

**Figure 6:**
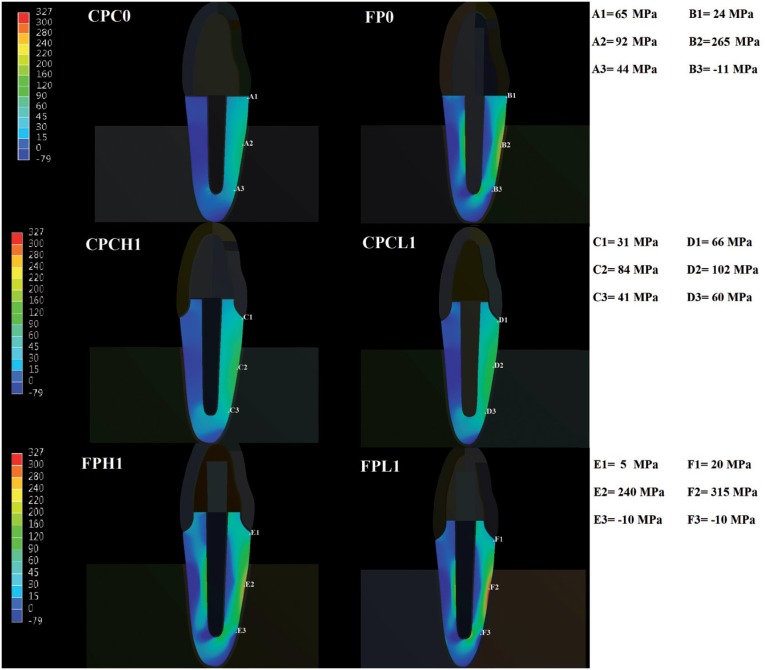
The images above shows: higher values of tensile stress at external portion of the root, in cervical and median third, in groups restored with fiber post (Fiber Post - FP0, Fiber Post higher than 1 mm - FPH1, Fiber Post lower than 1 mm - FPL1); higher values of tensile stress at apical third of root, in specimens restored with cast post and core (Cast Post and Core - CPC0, Cast Post and Core higher than 1 mm - CPCH1, Cast Post and Core lower than 1 mm - CPCL1) in comparison with roots restored with fiber posts

**Figure 7:**
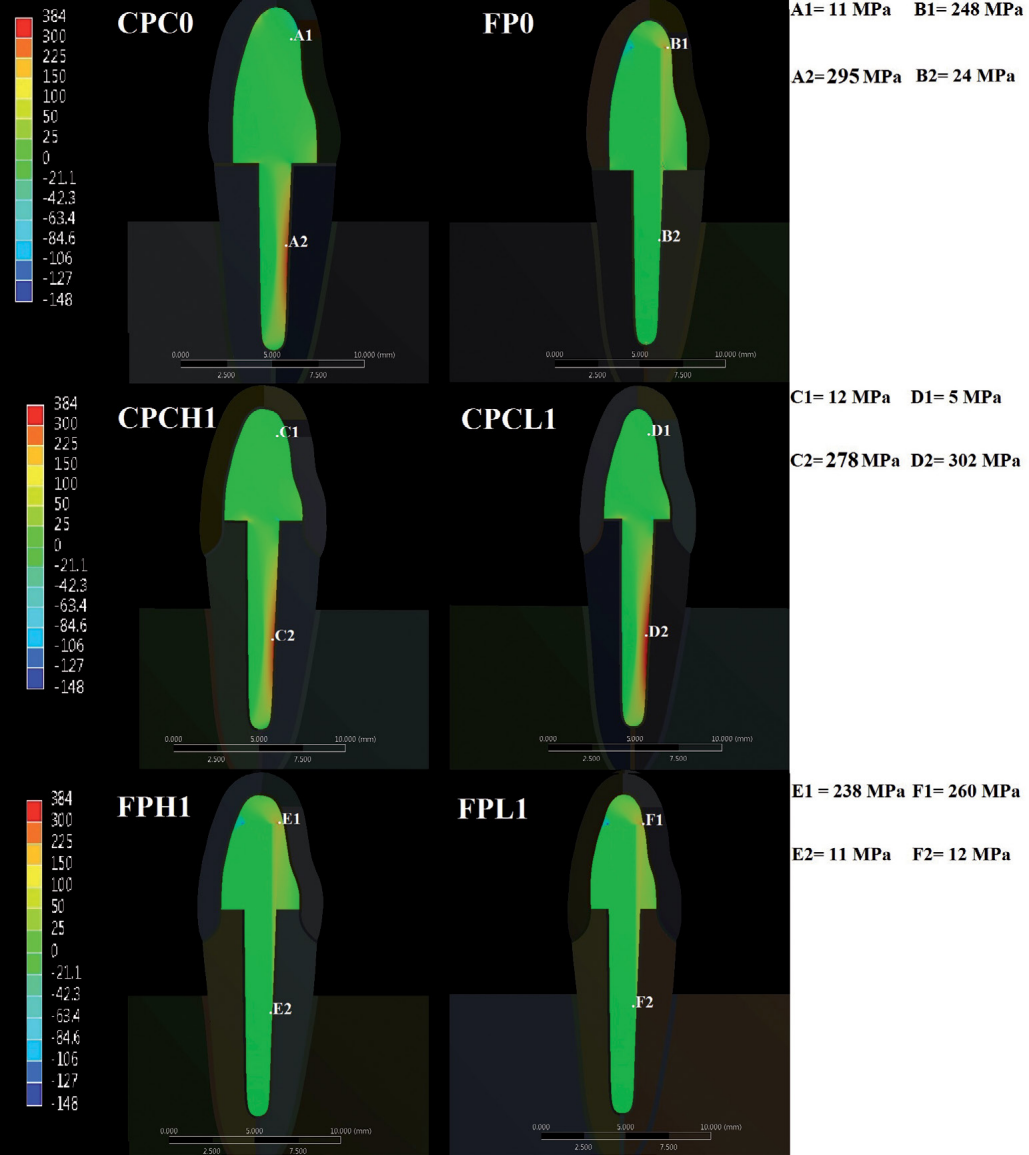
Images above show: higher values of tension stress at resin composite cores, in comparison with groups restored with cast post and cores (Fiber Post - FP0, Fiber Post higher than 1 mm - FPH1, Fiber Post lower than 1 mm - FPL1); higher values of tensile stress at internal root portion in groups restored with cast post and cores, in comparison with groups restored with fiber post (Cast Post and Core - CPC0, Cast Post and Core higher than 1 mm - CPCH1, Cast Post and Core lower than 1 mm - CPCL1)

The analysis showed a greater concentration of tensile stress in the groups with thickness of remaining coronal structure lower than 1 mm, compared with the groups with thickness higher than 1 mm (Figure 6). Moreover, the specimens restored with CPC concentrated the greatest tensile value in the apical region of the root (Figure 6) and inside root canal (Figure 7), while the teeth with glass fiber post had higher concentration of tensile values in the composite core (Figure 7) and in the middle third of the root, at the outer portion (Figure 6).

## Discussion

Based on our findings, we can notice that all remaining coronal structure and thickness of this structure influenced on the outcomes. Thus, the assumed null hypothesis was rejected at all levels.

As highlighted finding, the absence of remaining tooth structure lead to statistically lower survival rate values, regardless of the restorative strategy used. Despite the absence of *in vitro* studies evaluating the survival values, those results agree with the clinical study by Ferrari and collaborators^8^ (2012), which showed unfavorable prognosis for teeth without remaining coronal structure compared with those which had coronal structure. Additionally, loads to fracture were significantly lower for teeth without remaining structure (Table 1), which also corroborates with some *in vitro* studies^2^ ^,^ ^6^ ^,^ ^11^. This behavior may be explained by the worst stress distribution to the root structure, concentrating higher tensile values and providing gaps into the assembly, as observed in the test of finite elements (Figures 5, 6 and 7). Furthermore, in the groups with fiber post, the absence of remaining tooth structure leads to a larger quantity of restorative material in the core portion, which may generate defects and failures at the core.

Regarding the influence of the remaining dentin thickness in the coronal part on the load to fracture, statistical analysis showed that groups of remaining coronal thickness lower than 1 mm had loads to fracture lower than groups which had remaining coronal thickness greater than 1 mm and similar to specimens with no coronal structure (Table 1). These results may be related to the fact that these specimens focus higher tensile values in the root region, as shown by finite element analysis (Figure 5). This higher concentration may be due to the fact that these specimens have dimensions inferior than the other, thus having a higher risk of fracture.

Based on the survival analysis, both restorative options (cast post and fiber post) had similar behaviors (Table 1). This finding is consistent with clinical trial findings^18^, which concluded that both glass fiber and cast metal posts showed good and similar clinical performance. Additionally, we can notice that the failure mode observed was also consistent to clinical studies available, wherein the irreversible fracture was more frequent in tooth restored with cast metal core; while debonding of the retainer was the most common cause of failure in tooth restored with glass fiber posts^18^.

The load to fracture values were also similar between the strategies (cast post and core *vs* fiber post) (Table 1), agreeing with other studies that showed that both restorative strategies could be interesting regarding the biomechanical aspect^19^.

Although the similar values, the failure modes observed were different (Table 1), agreeing with other studies^6^ ^,^ ^7^ ^,^ ^16^ ^,^ ^23^, which may be due to different stress distribution in the specimens (Figure 6 and 7). Higher percentage of root fractures for the cast post and cores group may have been mainly caused by stress concentration in the inner portion of the root (Figure 7), as also observed by other study^19^, while lower tensile stress can be observed at the coronal portion of the tooth restored with fiber post (Figure 6). It can be explained considering that in a rigid body subjected to forces, the stress will concentrate on the structure created with higher stiffness and greater intensity will be transferred to the adjacent structure (Figure 7).

In groups restored with fiber posts, we observed a high concentration of tensile forces in the middle third of the root (Figure 6). However, we could also observe high tensile values in the composite core portion (Figure 7). Thus, as already mentioned by other studies^13^ ^,^ ^19^, the risk of radicular fractures in this scenario is smaller, since the tendency is that the post or core composite fail before the root break. In fact, this study has depicted a higher number of “debonding crown” for groups restored with fiber post (Table 1).

Specimens submitted to loading at 45° suffer influence of tensile, compression^3^ ^,^ ^23^ and shear^23^ stresses that may damage the restoration. The force in 45° is formed by a horizontal component that promotes compressive loading and a vertical component that produces bending of the restorative assembly inducing the formation of tensile stress on the buccal surface and compression on the lingual surface, besides generating shear stress inside (center) the structure. Tensile and compression stresses are maximum in the external portions and minimum in the center, differently from the shear stress that is maximum in the center and minimum in the outer portions^4^ ^,^ ^23^.

Previous studies^23^ ^,^ ^24^ that evaluated failures in roots restored with posts showed that a sequence of events occurs until the final failure. This fact repeated in this study to both groups with and without remaining coronal structure. For groups without remaining structure (Figures 5A-B), the failures were firstly adhesive on the lingual surface at the interface crown/root with displacement of the crown/core/post assembly (due to maximum tensile stress). After that, the retainer was loosened in the canal, being no longer a unique structure, the predominant stress was compressed and the failure evolved to total radicular fracture on the buccal surface (due to maximum compression stress). Besides, cracks and adhesive failures were observed on the interface dentine/ cement/post due to shear stress^23^.

The presence of ferrule modified the failure pattern, since the remaining coronal structure acts as the lever arm, probably hampering the fracture/failure progression. This explains the lower survival and load to fracture values of the groups without ferrule in our study. In the groups with remaining coronal structure, the presence of ferrule effect and its thickness had some variations in the internal failure pattern of the restorative assembly. In specimens with thickness lower than 1 mm, the displacement of the crown/core/ post assembly promoted firstly an adhesive failure in the lingual interface crown/root (due to tensile stress). Subsequently, it evolved to a crack on the lingual surface of the remaining coronal structure (for some specimens - Figures 5C-D) toward the inner part of the root canal and progressed to dentine/cement/post interface (due to shear stress). The crack possibly surrounded the posts, reaching the buccal wall of the root and achieving totally or partially the external root surface (due to compression stress) (Figures 5C, D, E, F). Differently, in the groups with remaining thickness greater than 1 mm, the evolution of the failures was similar, however the cracks in the lingual remaining structure were not observed, probably due to higher thickness of the ferrule (Figures 5G-H).

Even being a laboratory work, some care was taken aiming the quality and reliability of the study using procedures such as blinding the assessors, conducting sample calculation, randomization, among others^13^. In addition, aging by mechanical cycling allows to have a notion of system behavior predictability, because most closely, the conditions of intra-oral environment for reproducing tilt protocols, humidity, temperature and strength are similar to that observed *in vivo.* On the other hand, it is known that load to fracture tests (monotonic tests) do not simulate the exact condition that occurs in the mouth, and the execution of a clinical study evaluating the outcomes might be interesting to evaluate the clinical survival of teeth with different clinical condition. Similarly, although the analysis of the finite element results agrees with the observed results, performing a dimensional analysis will further enrich the findings.

## Conclusion

1. Specimens without remaining coronal structure had survival rates lower than teeth with remaining coronal structure, considering the same restorative strategies;
2. Specimens with remaining coronal structure with thickness greater than 1 mm support higher load values for fracture, despite of restorative strategy used;
3. Specimens with remaining coronal structure with thickness lower than 1 mm support the same load to fracture to the specimens without remaining coronal structure, regardless of restorative strategy used;
4. Specimens restored with fiber posts tend to suffer less catastrophic fracture compared with the cast post and core in the specimens with 2 mm height of the remaining coronal structure and thickness higher than 1 mm, allowing restoration of the new set.

## References

[1] Adebayo OA, Burrow MF, Tyas MJ (2008). Bond strength test: role of operator skill. Aust Dent J..

[2] Al-Omiri MK, Mahmoud AA, Rayyan MR, Abu-Hammad O (2010). Fracture resistance of teeth restored with post-retained restorations: an overview. J Endod..

[3] Assif D, Gorfil C (1994). Biomechanical considerations in restoring endodontically treated teeth. J Prosthet Dent..

[4] Beer FP, Johnston R (2015). Vector mechanics for engineers: statics.

[5] Belli S, Erdemir A, Yildirim C (2006). Reinforcement effect of polyethylene fibre in root-filled teeth: comparison of two restoration techniques. Int Endod J..

[6] Broch J, Marchionatti AM, Bergoli CD, Valandro LF, Kaizer OB (2015). Fracture resistance of weakened roots restored with different intracanal retainers. Gen Dent..

[7] Dietschi D, Romelli M, Goretti A (1997). Adaptation of adhesive posts and cores to dentin after fatigue testing. Int J Prosthodont..

[8] Ferrari M, Vichi A, Fadda GM, Cagidiaco MC, Tay FR, Breschi L (2012). A randomized controlled trial of endodontically treated and restored premolars. J Dent Res..

[9] Ferreira FV, Della Serra O (1981). Anatomia dental..

[10] Juloski J, Fadda GM, Monticelli F, Fajó-Pascual M, Goracci C, Ferrari M (2014). Four-year survival of endodontically treated premolars restored with fiber posts. J Dent Res..

[11] Juloski J, Radovic I, Goracci C, Vulicevic ZR, Ferrari M (2012). Ferrule effect: a literature review. J Endod..

[12] Mamoun JS (2014). On the ferrule effect and the biomechanical stability of teeth restored with cores, posts, and crowns. Eur J Dent..

[13] Montenegro R, Needleman I, Moles D, Tonetti M (2002). Quality of RCTs in periodontology - a systematic review. J Dent Res..

[14] Roscoe MG, Noritomi PY, Novais VR, Soares CJ (2013). Influence of alveolar bone loss, post type, and ferrule presence on the biomechanical behavior of endodontically treated maxillary canines: strain measurement and stress distribution. J Prosthet Dent..

[15] Sagsen B, Zortuk M, Ertas H, Er O, Demirbuga S, Arslan H (2013). *In vitro* fracture resistance of endodontically treated roots filled with a bonded filling material or different types of posts. J Endod..

[16] Santos AF, Meira JB, Tanaka CB, Xavier TA, Ballester RY, Lima RG (2010). Can fiber posts increase root stresses and reduce fracture?. J Dent Res..

[17] Santos PC, Veríssimo C, Soares PV, Saltarelo RC, Soares CJ, Marcondes Martins LR (2014). Influence of ferrule, post system, and length on biomechanical behavior of endodontically treated anterior teeth. J Endod..

[18] Sarkis-Onofre R, Jacinto RC, Boscato N, Cenci MS, Pereira-Cenci T (2014). Cast metal vs. glass fibre posts: a randomized controlled trial with up to 3 years of follow up. J Dent..

[19] Scotti R, Ferrari M, Baldissara P (2003). Pinos de fibra considerações técnicas e aplicações clínicas. Propriedades mecânicas e avaliação *in vitro*.

[20] Silva NR, Raposo LH, Versluis A, Fernandes AJ, Soares CJ (2010). The effect of post, core, crown type, and ferrule presence on the biomechanical behavior of endodontically treated bovine anterior teeth. J Prosthet Dent..

[21] Soares JC, Santana FR, Castro CG, Santos PC, Soares PV, Qian F (2008). Finite element analysis and bond strength of a glass post to intrarradicular dentin: comparison between microtensile and pushout tests. Dent Mat..

[22] Versluis A, Messer HH, Pintado MR (2006). Changes in compaction stress distributions in roots resulting from canal preparation. Inter Endod Jour..

[23] Wandscher VF, Bergoli CD, Limberger IF, Ardenghi TM, Valandro LF (2014). Preliminary results of the survival and fracture load of roots restored with intracanal posts:weakened vs nonweakened roots. Oper Dent..

[24] Wandscher VF, Bergoli CD, Limberger IF, Pereira-Cenci T, Baldissara P, Valandro LF (2016). Fractographical analysis and biomechanical considerations of a tooth restored with intracanal fiber post: report of the fracture and importance of the fibers arrangements. Oper Dent In Press..

[25] Watanabe MU, Anchieta RB, Rocha EP, Kina S, Almeida EO, Freitas AC (2012). Influence of crown ferrule heights and dowel material selection on the mechanical behavior of root-filled teeth: a finite element analysis. J Prosthodont..

[26] Wiskott HW, Nicholls JL, Belser UC (1995). Stress fatigue: basic principles and prosthodontic implications. Int J Prosthodont..

